# (Es)ketamine as a treatment for depressive episodes with psychotic features: systematic review

**DOI:** 10.1192/bjo.2025.10830

**Published:** 2025-10-14

**Authors:** Henrique Castro Santos, Alexandra Rodrigues, Tiago Machado, Manuel Gonçalves-Pinho, Allan H. Young, Mario F. Juruena

**Affiliations:** Department of Psychological Medicine, Institute of Psychiatry, Psychology and Neuroscience, King’s College London, London, UK; South London and Maudsley NHS Foundation Trust, Bethlem Royal Hospital, London, UK; Júlio de Matos Hospital, São José Local Health Unit, Lisbon, Portugal; Neuroradiology Department, São José Local Health Unit, Lisbon, Portugal; Neuroradiology Department, SESARAM, EPERAM, Funchal Central Hospital, Funchal, Portugal; Laboratory of Clinical Pharmacology and Therapeutics, Faculty of Medicine, University of Lisbon, Lisbon, Portugal; CINTESIS@RISE, Department of Clinical Neurosciences and Mental Health, Faculty of Medicine, University of Porto, Porto, Portugal; Department of Psychiatry and Mental Health, Tâmega e Sousa Local Health Unit, Penafiel, Portugal; Department of Brain Sciences, Imperial College London, London, UK

**Keywords:** Ketamine, psychotic, depressive disorders, bipolar type I or II disorders, esketamine

## Abstract

**Background:**

Psychotic symptoms in depression are linked to worse outcomes, and treatment options are limited. Ketamine and esketamine are effective antidepressants, yet most studies have excluded patients with a history of psychotic symptoms.

**Aims:**

To evaluate by systematic review the efficacy and safety of ketamine and esketamine in treating patients with unipolar or bipolar depressive episodes with psychotic features.

**Method:**

A comprehensive search of the PubMed, Ovid and Web of Science databases was conducted up to 2 November 2023. We included any study that reported the use of ketamine or esketamine in patients with depressive episodes with psychotic symptoms. The primary outcomes assessed were variations in depressive and psychotic symptoms and the incidence of adverse events. The protocol was preregistered in PROSPERO (CRD42023488524).

**Results:**

Ten studies were included, encompassing 60 patients with unipolar depression with psychotic symptoms and 19 patients with bipolar depression with psychotic symptoms. Treatment with (es)ketamine showed mean score changes on the Montgomery–Åsberg Depression Rating Scale ranging from −13.7 to −18.2 points in open-label studies of patients with unipolar depression with psychotic symptoms. Up to 50% of participants achieved remission. The largest study with patients with bipolar depression with psychotic symptoms reported a mean Montgomery–Åsberg Depression Rating Scale score change of −14.9 points. Adverse events were mostly mild and transient. There were no reports of switches to (hypo)mania or deterioration of psychotic symptoms, and in six studies there was substantial improvement of the latter.

**Conclusions:**

The available evidence suggests that (es)ketamine shows antidepressant effects in patients with depressive episodes with psychotic features and has a reasonable safety profile. However, the heterogeneity of the studies included in this review and the high risk of bias warrant caution in interpreting the findings and underscore the need for further trials to confirm these preliminary results.

Depressive and bipolar disorders are major causes of disability.^1^ Psychotic symptoms have been found in 28% of patients with unipolar depression, translating to a prevalence of 0.5%.^2^ However, these numbers might be lower in the general population, as suggested by an European epidemiologic study (*n* = 18 960) which found that 18.5% of those with major depressive episodes had psychotic features, corresponding to an overall prevalence of 0.4%.^3^ The presence of psychotic symptoms in the context of unipolar depression has been associated with worse outcomes, namely higher mortality, lower functioning, higher numbers of suicide attempts and hospital admissions, and longer in-patient treatment, reflecting a more severe form of the disorder.^2,4,5^ In a study of bipolar depression, 20% of individuals with a bipolar depressive episode in an in-patient setting exhibited psychotic symptoms.^6^ Despite increasing numbers of studies on this disorder, there is still a lack of evidence on the outcomes and treatment of patients with bipolar depression with psychotic symptoms (BPwP).^7,8^

Up to 30% of patients suffering from major depression do not achieve symptom remission, and 30% of these do not show any response to treatment.^9^ There is little evidence on treatment resistance in patients with bipolar depressive episodes, but more than one-third of such patients fail to respond to multiple medication trials.^10–13^ Current pharmacological treatments for both conditions are suboptimal. Even when effective, they are limited by the fact that a duration of several weeks is required to achieve response.^14–16^ Furthermore, specific treatment options for psychotic depression are heavily under-studied.^17^

Multiple randomised controlled trials (RCTs) have demonstrated rapid and effective antidepressant effects of ketamine and esketamine in both unipolar and bipolar depression, including treatment-resistant cases, as reported in a recent meta-analysis.^18^ This was reflected in the inclusion of intranasal esketamine and intravenous (i.v.) ketamine as second-line treatments for difficult-to-treat depression in the most recent Canadian Network for Mood and Anxiety Treatments guidelines for management of major depressive disorder in adults.^19^ In addition, ketamine has been shown to be particularly effective in reduction of suicidal ideation in both bipolar and unipolar depression.^20,21^

Ketamine is a racemic mixture that includes equal amounts of S-ketamine (esketamine) and R-ketamine (arketamine) enantiomers. Proposed mechanisms underlying the antidepressant effects of ketamine’s enantiomers include glutamate release via *N*-methyl-d-aspartic acid antagonism at gamma-aminobutyric acid interneurons with downstream release of brain-derived neurotrophic factor and increased synaptogenesis. These and other proposed mechanisms have been extensively reviewed.^22^ Ketamine has been studied in animal models of drug-induced schizophrenia owing to its psychotomimetic effects.^23^ Evidence from a meta-analysis and a case series showed that administration of ketamine to patients with schizophrenia and healthy volunteers increased psychotic symptoms.^24,25^ This potential to exacerbate psychotic symptoms has motivated the exclusion of patients with unipolar or bipolar depression with a history of psychotic symptoms from most of the trials exploring the antidepressant effects of ketamine and esketamine. We found one previous systematic review^26^ that included studies that assessed the effects of (es)ketamine in patients with a history of past or current psychotic symptoms. The review concluded that short-term ketamine treatment may be safe and effective in patients with depressive symptoms in the context of diagnoses ranging from unipolar and bipolar depression to schizoaffective disorder and schizophrenia. However, the findings were based on few, small and limited studies. Since then, more studies have been published involving patients with depressive episodes with current psychotic symptoms. In this systematic review, we aimed to assess more comprehensively the efficacy and safety of ketamine and esketamine in patients with a depressive (unipolar or bipolar) episode with psychotic symptoms.

## Method

This study is reported in accordance with the Preferred Reporting Items for Systematic Reviews and Meta-Analyses (PRISMA)^27^ statements. PRISMA checklists can be found in Supplementary Tables 1 and 2 available at https://doi.org/10.1192/bjo.2025.10830. The study protocol was preregistered and is available from: https://www.crd.york.ac.uk/prospero/display_record.php?ID=CRD42023488524. Only one change was made to the protocol on 11 December 2023; this included a more detailed description of the strategy for data synthesis.

### Search strategy and selection criteria

A systematic search was performed to identify relevant articles, and a two-step literature search was conducted by two independent researchers (A.R. and H.C.S.). The PubMed, Ovid and Web of Science (Clarivate Analytics) databases were searched from inception until 2 November 2023. The Web of Science Core Collection (1900 to –present), KCI-Korean Journal Database (1980 to present), MEDLINE (1950 to present), Preprint Citation Index (1991 to present), ProQuest Dissertations & Theses Citation Index (1637 to present) and SciELO Citation Index (2002 to present) were consulted as part of the systematic screening, as well as the Cochrane Central Register of Controlled Trials and the Ovid PsycINFO (1806 to October week 4 2023) and Embase (1974 to 2 November 2023) databases. The following search strategy was applied: (‘ketamine’ OR ‘esketamine’ OR ‘*ketamine’) AND (‘depress*’ OR ‘bipolar’) AND (‘psychosis’ OR ‘psychotic’). Retrieved articles were screened by A.R. and H.C.S. as abstracts. After exclusion of articles not meeting the inclusion criteria, the full texts of the remaining studies were assessed for eligibility. We completed the search process by manually reviewing the references of previously published articles and retrieving any additional relevant papers. In case of disagreement, consensus was sought through discussion.

Studies were included if they: (a) were individual studies; (b) were conducted in a sample that included individuals who met criteria for a depressive episode with psychotic symptoms according to psychometric instruments or clinical criteria and were treated with ketamine or esketamine; (c) provided longitudinal data on efficacy or adverse events; and (d) were published in English. Case series and case reports were included given the absence of RCTs and overall lack of studies on this topic found in a preliminary search. Studies were excluded if they were: (a) reviews, abstracts, conference proceedings or study protocols; (b) studies not including individuals with a diagnosis of a depressive episode; (c) studies in which ketamine was provided simultaneously with electroconvulsive therapy (ECT); (d) studies conducted in animals; (e) published in a language other than English.

### Data extraction

Two researchers (A.R. and H.C.S.) independently extracted data from the included studies into a Microsoft Excel spreadsheet. Data extraction started on 6 January 2024. Discrepancies were resolved through consensus with other researchers (M.J. and T.M.).

The following variables were extracted: first author and year of publication; country; type of study; sample size; age as mean and s.d. (range) in years; percentage females; method used to confirm the diagnosis of depressive episode with psychotic symptoms; diagnosis of interest (unipolar or bipolar depressive episode); psychiatric comorbidities (percentage substance use disorders, percentage anxiety disorders, percentage psychotic disorders, others); mean dose and posology of ketamine and/or esketamine; formulation of ketamine and/or esketamine administered; medication received (percentage antidepressants, percentage antipsychotics, percentage mood stabilisers, percentage benzodiazepines); mean percentage and/or absolute score variation in depressive symptoms; mean percentage and/or absolute score variation in psychotic symptoms; mean percentage and/or absolute score variation in anxiety symptoms; time points for the different outcomes; follow-up period; mean percentage and/or absolute number of adverse events reported; mean percentage and/or absolute number of severe adverse events; time to adverse event remission; and discontinuation due to adverse events (absolute number and percentage).

### Risk of bias and quality assessment

RCTs were assessed using the Cochrane risk-of-bias tool for RCTs (RoB2).^28^ Cohort studies were assessed using the Newcastle–Ottawa Scale. Studies were awarded a maximum of eight stars on items related to representativeness, exposure, outcomes, follow-up period and loss to follow-up. Given the lack of tools for assessment of the methodological quality of case reports and case series, we derived items from the Newcastle–Ottawa scale^29^ that were appropriate for this systematic review, as in previous reviews.^30^ Accordingly, five items were used with a ‘yes’ or ‘no’ answer to indicate whether the item was suggestive of poor methodological quality or not. For each positive answer, a point was awarded, and the qualitative assessment was as follows: good if five points; moderate if four points; poor if three points or fewer. In addition, we evaluated case reports and case series with the Joanna Briggs Institute Critical Appraisal Checklists for Case Reports and Case Series, respectively.^31,32^

The same two reviewers (A.R. and H.C.S.) independently assessed the methodological quality of the included studies. In case of disagreement, consensus was sought through discussion.

### Strategy for data synthesis

Findings were systematically synthesised regarding the efficacy and safety of ketamine and esketamine in the population of interest. Summaries of these findings are presented in tables. The small sample and high heterogeneity of the methodologies used in the included studies meant they were not suitable for high-quality meta-analysis or statistical synthesis. Available efficacy outcomes are presented as mean changes in scores and rates of response and remission. For easier comparison of the antidepressant effects between studies which used different instruments to assess depressive symptoms, we converted mean score changes to percentages and presented these calculated data in separate table columns. Adverse events are presented as absolute incidence numbers, and psychotic symptoms are shown as mean score changes. For both efficacy and safety, outcomes were compared across studies for each condition (unipolar and bipolar depressive episodes with psychotic symptoms).

## Results

**Fig. 1:**
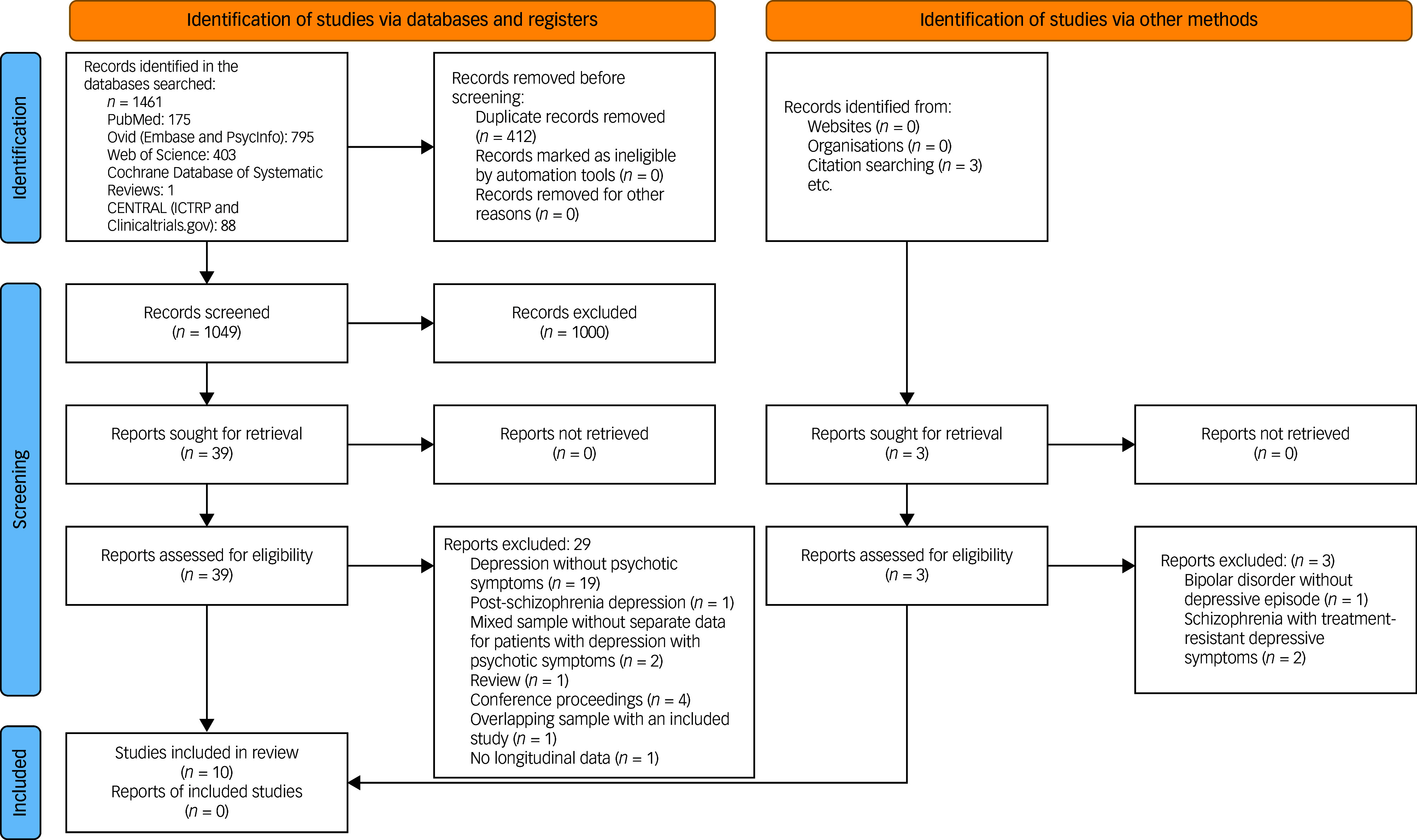
Preferred Reporting Items for Systematic Reviews and Meta-Analyses (PRISMA) diagram for the systematic review. ICTRP, International Clinical Trials Registry Platform.

Overall, 1461 articles were identified through the database search, and three additional records were retrieved by manually searching references (Fig. 1). After removal of duplicates, 1049 unique articles were assessed through title and abstract screening. Of these, 49 full-text articles were assessed for eligibility. Ten articles were included in the final review and encompassed one open-label RCT, two retrospective cohort studies, one case series and six case reports. A summary of the characteristics of these studies can be found in Table 1. The PRISMA diagram is presented in Fig. 1.

**Table 1:** Summary of characteristics of the included studies

Study	Type of study	*N* ^a^	Diagnosis	Age, years	Number of females (%)	Intervention	Follow-up duration
Ekstrand et al, 2022	Open-label randomised trial	32/186	UDwP (*N* = 32)	Ketamine group: 55 ± 18 (18–84)(*n* = 95); ECT group: 50 ± 18 (20–85) (*n* = 91); N/D for the subgroup	119 (64% of 186)	i.v. ketamine 0.5 mg/kg; three times per week (*n* = 18/32) or ECT three times per week during up to 4 weeks (*n* = 14/32)	Up to 4 weeks in the *post hoc* analysis; up to 12 months of follow-up for remitters
Souza-Marques, et al, 2022	Retrospective chart review with a matched case–control analysis	15/45	UDwP (*n* = 15) and TRUD (n = 30)	UDwP: 52.1 (s.d. 18.7); TRUD: 47.7 (s.d. 13.7)	UDwP: 6 (40%) TRUD: 15 (50%)	UDwP: i.v. or s.c. esketamine 0.5 mg/kg single dose; TRUD: i.v. esketamine 0.25 mg/kg single dose	24 h
Gałuszko-We¸gielnik et al, 2023	Observational, retrospective, uncontrolled	35/35	TR UDwP (n = 17)	TR UDwP: 51.2 (s.d. 11.1)	9 (52.9%)	i.v. ketamine 0.5 mg/kg; two times per week for 4 weeks	5 weeks
			TR BDwP (n = 18; BPI = 12; BPII = 6)	TR BDwP: 46.3 (s.d. 17.4)	12 (66.7%)		
Ajub et al, 2017	Case series	3/4	UDwP (Patients 1 and 2); BDwP with mixed features (Patient 3)	Patient 1: 41; patient 2: 45; patient 3: 44;	3 (100%)	Esketamine 0.5 mg/kg single dose i.v. (patient 1) or s.c. (patients 2 and 3)	Patients 1 and 3: 2 weeks; patient 2: 4 weeks
Mischell et al, 2018	Case report	2/2	UDwP (*n* = 2)	Patient 1: 81; patient 2: 77	0	i.v. Ketamine; patient 1: 0.4 mg/kg/h for 24 h, paused for 16 h, restarted 0.3 mg/kg/h for 48 h; patient 2: 0.3 to 0.4 mg/kg/h for 1 week	Patient 1: 3 months; patient 2: unspecified number of ‘weeks’ until death from cancer
da Frota Ribeiro et al, 2016	Case report	2/2	Patient 1 TR UDwP Patient 2 UDwP	Patient 1: 52; patient 2: 55	2 (100%)	i.v. ketamine 0.5 mg/kg; patient 1: three sessions; patient 2: five sessions	N/D
Trejos Orozco et al, 2022	Case report	1/1	UDwP and catatonic features	63	1 (100%)	i.v. ketamine 0.5 mg/kg, two administrations 4 days apart	1 week
Carter et al, 2022	Case report	1/1	TR UDwP	28	1 (100%)	Intranasal esketamine (56 mg for the first two treatments; 84 mg for the remaining): two times per week for 4 weeks; once per week for 4 weeks; two times per month for 1 month	9 months
Veraart et al, 2021	Case report	1/1	TR UDwP	55	1 (100%)	Oral esketamine 0.5–2.0 mg/kg, two times per week for 18 months	18 months
Zarrinnegar et al, 2019	Case report	1/1	TR UDwP	15	1 (100%)	i.v. ketamine 0.5 mg/kg on days 3, 5, 7, 10, 17 and 24	’Several months’

BDwP, bipolar depression with psychotic symptoms; BPI, bipolar type I; BPII, bipolar type II; ECT, electroconvulsive therapy; i.v., intravenous; N/D, not disclosed; s.c., subcutaneous; TR, treatment resistant; TRUD, treatment-resistant unipolar depression; UDwP, unipolar depression with psychotic symptoms.

a Sample number details: number of individuals with the diagnosis of interest/total sample.

The small sample (10 studies, 79 patients) and high heterogeneity of methodologies meant the included reports were not suitable for high quality meta-analysis. In particular, the studies varied in design, (es)ketamine formulation, dosage regimens and assessment time points. Therefore, we provide a summary of outcomes regarding variation of depressive symptoms (Table 2), psychotic symptoms and occurrence of adverse events (Table 3). Supplementary Tables 3 and 4 present the quality assessment and risk of bias of the included studies. Supplementary Table 5 shows additional data extracted from the studies, namely prevalence of comorbidities and concurrent medication.

**Table 2:** Summary of efficacy outcomes

Study	Diagnosis	Depressive symptoms					
		Mean absolute variation (s.d.)	Mean change, %^a^	Mean change	Response^b^	Remission	Clinician-observed and patient-reported change
Ekstrand et al, 2022	UDwP (*n* = 32)	MADRS baseline: ketamine 37.6 ± 6.1 *v*. ECT 37.1 ± 4.6 (*P* = 0.79); MADRS post-treatment: ketamine 19.4 ± 16.7 *v*. ECT 10.1 ± 11.1 (*P* = 0.069, Cohen d = 0.68)	MADRS change in up to 4 weeks: ketamine −48.4% (s.d. 71.4%; *P* < 0.001); ECT −74.9% (s.d. 42.4%; *P* < 0.001)	MADRS change in up to 4 weeks: ketamine −18.2 (s.d. 13.0; *P* < 0.001); ECT −27.8 (s.d. 11.8; *P* < 0.001)	N/D	In up to 4 weeks:^d^ ketamine 9/18 (50%) *v.* ECT 11/14 (79%) (*χ* ^2^ = 2.7, *P* = 0.15, odds ratio: 0.27 [CI 0.056, 1.3])	N/D
Souza-Marques et al, 2022	UDwP (*n* = 15) TRUD (*n* = 30)	Baseline MADRS (s.d.): 40.9 (8, 33) in the UDwP group *v*. 33.6 (5.36) in the TRUD group (*P* = 0.004). Clinically meaningful^c^ change at 24 h: 10 (66%) in the UDwP group *v*. 22 (73%) in the TRUD group Worsening of depressive symptoms at 24 h: 2 (13%) of UDwP group *v*. 2 (6%) of TRUD group			5 (33%) of UDwP group 12 (40%) of TRUD group (including remissions)	At 24 h^e^ 2 (13%) in the UDwP group, 7 (23%) in the TRUD group	N/D
Gałuszko-We¸gielnik et al, 2023	TR UDwP (*n* = 17)	MADRS baseline: 29.4 (5.0) MADRS week 5: 15.7 (6.6)	MADRS change at week 5 = −46.6% (s.d. 60.4%)	MADRS change at week 5 = −13.7 (s.d. 8.28)	N/D	N/D	N/D
	TR BDwP (*n* = 18)	MADRS baseline: 30,6 (5.7); week 5: 15.7 (6.3)	MADRS change week 5 = −48,7% (s.d. 57.0%)	MADRS change week 5 = −14.9 (s.d. 8.50)	N/D	N/D	N/D
Ajub et al, 2017	UDwP (patients 1 and 2); BDwP (patient 3)	MADRS pre-treatment and 24 h post-treatment: patient 1, 55, 16; patient 2, 36, 2; patient 3, 42, 7 MADRS mean score pre-treatment^a^: 44.3 (s.d. 9.7); MADRS mean score post-treatment^a^: 8.3 (s.d. 7.1)	Change at 24 h Mean change −81.3% (s.d. 7.4%; *P* = 0.109^f^) Patient 1 change: −70.9%; patient 2 change: −94.4%; patient 3 change: −83.3%	Change at 24 h Mean change^a^ −36.00 (s.d. 2.65; *P* = 0.109^f^) Patient 1 change: −39; patient 2 change: −34; patient 3 change: −35	100%^a^	2 (66.6%) at 24 h, of which patient 3 maintained remission at 2 weeks and patient 2 at 4 weeks	Patient 1: marked improvement at 24 h; ‘mild depressive symptoms’ at 2 weeks; patient 2: remission at 24 h and 2 weeks; patient 3 reported remission at 24 h and 2 weeks
Mischell et al, 2018	UDwP (*n* = 2)	No instrument was used to assess depressive symptoms. Patient 1 reported depressive symptoms decrease after ∼2 days; patient 2: ‘affect and personal engagement improved significantly’					
da Frota Ribeiro et al, 2016	Patient 1 TR UDwP Patient 2 UDwP	Time points not specified Patient 1: HRSD from 19 to 9; Patient 2: HRSD from 29 to 8	Time points not specified Patient 1 change: −52.6%; patient 2 change: −72.4%	Patient 1 change: −10; patient 2 change: −21	100%^a^	0%^e^	’Dramatic’ mood improvement in both cases
Trejos Orozco et al, 2022	UDwP and catatonic features (*n* = 1)	No instrument was used to assess depressive symptoms					Persistence of depressive symptoms but improvement of catatonic symptoms at 1 week
Carter et al, 2022	TR UDwP (*n* = 1)	QIDS scores before sessions: baseline, 17; second session, 18; third session, 15; ninth session, 9; 9 months follow-up, 8	QIDS change at 4 weeks: −52.9%; QIDS change at 9 months: −47.1%	QIDS change at 4 weeks: −8; QIDS change at 9 months: −9	100% response at 4 weeks but not at 9 months^a^	0%^e^	Improvement of mood, anhedonia, suicidal ideation and anxiety. Remission of auditory hallucinations, and improved insight and intensity regarding visual hallucinations
Veraart et al, 2021	TR UDwP (*n* = 1)	IDS-SR baseline scores: 54; HRSD 24 IDS-SR scores at 6 weeks: 30; HRSD 6	IDS-SR change at 6 weeks: −44.4% HRSD change at 6 weeks: −75.0%	IDS-SR change at 6 weeks: −24 HRSD change at 6 weeks: −18	100% (*n* = 1) response at 6 weeks according to HRSD scores^a^	100% (*n* = 1) at 6 weeks	author reported remission at 18 months
Zarrinnegar et al, 2019	TR UDwP (*n* = 1)	MADRS: ∼31 (day 3), ∼30 (day 5), 32 (day 7), 22 (day 13), ∼21 (day 17); CDRS-R: ∼67 (day 0), ∼62 (day 3), ∼56 (day 5), ∼49 (day 13), ∼41 (day 17); ∼44 (day 54)^a^	MADRS change at 17 days: −45.2% CDRS-R change at 17 days: −38.8%; CDRS-R change at 54 days: −34.3%	MADRS change at 17 days: −14 CDRS-R change at 17 days: −26; at 54 days: −23	0% response^a^	0%^e^	’[D]espite objective improvements in symptomatology, patient continued to subjectively deny improvement until 2 weeks after final infusion’. Authors reported absence of depressive symptoms at 1 month and ‘continued to do well during our follow-up of the ensuing several months’

BDwP, bipolar depression with psychotic symptoms; CDRS-R, Children’s Depression Rating Scale – Revised; ECT, electroconvulsive Therapy; HRSD, Hamilton Rating Scale for Depression; IDS-SR, Inventory for Depressive Symptomatology – Self Rated; MADRS, Montgomery–Åsberg Depression Rating Scale; N/D, not disclosed; TR, treatment resistant; UD, unipolar depression; UDwP, unipolar depression with psychotic symptoms; QIDS, Quick Inventory of Depressive Symptomatology.

a . Calculated on the basis of available data.

b . Response defined as a score reduction of at least 50%.

c . Clinically meaningful change was defined as a minimum decrease of 10 points after treatment. Overall variation or mean MADRS score at 24 h was not disclosed.

d . Remission was defined as a MADRS score ≤10 persisting over at least two subsequent treatment sessions or a minimum of 5 days.

e . Remission was defined as post-treatment MADRS score ≤9 or QIDS ≤5 or HRSD ≤7.

f . Wilcox-ranked test was used to compare the median differences between pre- and post-intervention scores.

**Table 3:** Adverse events and description of psychotic symptoms in the included studies

Study	Diagnosis	Psychotic symptoms			Adverse events		
		Percentage variation^a^	Absolute variation (s.d.)	Clinician-observed or patient-reported change	Any (*n*)	Severe (*n*)	Mean time to event remission, days
Ekstrand et al, 2022	UDwP (*n* = 32)	Modified BPRS was used, but individual scores were not reported		N/D	85/91 in the ketamine group versus 85/90 in the ECT group (*P* non-significant); N/D for the UDwP subgroup	ECT: 23/90; ketamine: 14/91; *P* = 0.09; N/D for the UDwP subgroup	Fatigue (17), sleep disturbance (6), muscle pain (5), constipation and/or diarrhoea (4), dissociation (2), salivation or dry mouth (2), vertigo (2), anxiety (1), affect lability (1), confusion (1), blurred vision (1), headache (1), nausea (1)
Souza-Marques et al, 2022	UDwP (*n* = 15) TRUD (*n* = 30)	No instrument was used to assess psychotic symptoms		N/D	Mild dissociative symptoms (*n* not disclosed)	N/D	Up to 2 h post-dose
Gałuszko-We¸gielnik et al, 2023	TR UDwP (*n* = 17)	BPRS-P^b^ change at week 5: −100% (30.6%)	BPRS-P (s.d.) baseline: 7.2 (2.2) Week 5: 0.0 (0.0) Mean change = −7.2 (2.2)	N/D	Dissociation: CADSS scores (peak 30 min after II infusion) TR UDwP up to 15.5 (15.0)	N/D	N/D
	TR BDwP (*n* = 18)	BPRS-P Change at week 5: −94.5% (43.5%)	BPRS-P (s.d.) baseline: 7.3 (2.6) Week 5: 0.4 (1.5)Mean change = −6.9 (3.0)	N/D	Dissociation: CADSS scores (peak 30 min after II infusion) TR BDwP up to 10.7 (8.5)	N/D	N/D
Ajub et al, 2017	UDwP (patients 1 and 2); BDwP (patient 3)	BPRS Mean change: −60.9% (SD 26.2%; *p* = 0.02^c^)	BPRS pre-treatment and 24 h post-treatment: patient 1, 53, 22; patient 2, 69, 24; patient 3, 49, 21. Mean change^a^: −34.7 (s.d. 9.1; *P* = 1.09^c^)	Patient 1: remission at 24 h and 2 weeks; patient 2: remission at 24 h and at 4 weeks; patient 3: remission at 24 h and at 2 weeks	Patient 1: dissociation, nausea, light-headedness; patient 2: dissociation	0	Patient 1: N/D; Patient 2: 2 h
Mischell et al, 2018	UDwP (*n* = 2)	No instrument was used to assess psychotic symptoms		Decreased psychotic symptoms	N/D	N/D	N/D
da Frota Ribeiro et al, 2016	Patient 1 TR UDwP patient 2 UDwP	No instrument was used to assess psychotic symptoms		Patient 1: remission ‘within hours’ of administration; patient 2: remission after administration	Patient 1: mild dissociation, fatigue, mild headache; patient 2: N/D	0	N/D
Trejos Orozco et al, 2022	UDwP and catatonic features (*n* = 1)	No instrument was used to assess psychotic symptoms		Catatonic features improved; persistence of psychotic symptoms at 1 week	N/D	N/D	N/D
Carter et al, 2022	TR UDwP (*n* = 1)	No instrument was used to assess psychotic symptoms		Remission of auditory hallucinations; reduction of visual hallucinations; insight achieved for the hallucinations	Mild nausea; mild headache in one session	0	N/D
Veraart et al, 2021	TR UDwP (*n* = 1)	No instrument was used to assess psychotic symptoms		Remission for at least 18 months	Transient dizziness	0	N/D
Zarrinnegar et al, 2019	TR UDwP (*n* = 1)	No instrument was used to assess psychotic symptoms		Authors reported absence of psychotic symptoms at 1 month and ‘continued to do well during our follow-up of the ensuing several months’	Derealisation and nausea (managed with chlorpromazine)	N/D	N/D


BDwP, bipolar depression with psychotic symptoms; BPRS, Brief Psychiatric Rating Scale; CADSS, Clinician-Administered Dissociative States Scale; ECT, electroconvulsive therapy; N/D, not disclosed; TR, treatment resistant; UD, unipolar depression; UDwP, unipolar depression with psychotic symptoms

a . Calculated on the basis of available data.

b . BPRS-P includes the following four items: suspiciousness, hallucinations, unusual thought content and conceptual disorganisation.

c . Wilcox-ranked test was used to compare the median differences between pre- and post-intervention scores.

In the included studies, a total of 60 patients with unipolar depression with psychotic symptoms (UDwP) and 19 patients with BDwP underwent treatment with either ketamine (*n* = 59) or esketamine (*n* = 20). Of these patients, 21 with UDwP and 18 with BDwP were considered as having treatment-resistant episodes. Treatment resistance was defined by Gałuszko-We¸gielnik et al^33^ and Souza-Marques et al^34^ studies as unsatisfactory response to two appropriate and sufficient treatment interventions. The four treatment-resistant patients described in case reports had unsatisfactory responses to multiple antidepressants,^35^ as well as to augmentation of antidepressants with antipsychotics and mood stabilisers,^36,37^ and in one case there was no significant improvement after ECT and deep brain stimulation.^38^ Patient age varied from 15 to 85 years.

Ketamine was administered i.v. in all cases at a dosage of 0.5 mg/kg per session, except in one of the case reports,^39^ where it was administered at a rate of 0.3 to 0.4 mg/kg/h for up to 1 week. Esketamine was provided in diverse formulations: i.v. or subcutaneous (s.c.) 0.5 mg/kg single dose;^34,40^ intranasal 56 to 84 mg;^37^ and oral 0.5 to 2.0 mg/kg.^38^ Administration frequency varied from a single dose to three doses per week for up to 1 month (ketamine) to 18 months (esketamine). Follow-up ranged from 24 h to 5 weeks after treatment in the larger studies, and up to 18 months in the case reports.

### Efficacy

The following two subsections focus on efficacy outcomes per diagnosis (unipolar and bipolar), which are summarised in Table 2. As anxiety symptoms were not specifically assessed by any of the included studies (although some studies used the Brief Psychiatric Rating Scale; BPRS), we present data for depressive symptoms.

#### Unipolar depression with psychotic symptoms

In the open-label trial^41^ and cohort study^33^ which used i.v. ketamine 0.5 mg/kg two to three times per week over 4 weeks, the mean score changes in Montgomery–Åsberg Depression Rating Scale (MADRS) after treatment were −13.7 (s.d. 8.28) and −18.2 (s.d. 13.0) points, respectively. In the latter study, 50% of the ketamine group achieved remission, compared with 79% in the ECT group (*P* = 0.15), in which the mean score reduction was −27.8 points (s.d. 11.8). However, despite equal frequency of sessions (3 times per week), the number of treatment sessions was slightly higher in the ECT group: 7.8 (s.d. 2.4; median 8) for ECT *v*. 6.8 (s.d. 3.3; median 6) in the ketamine group (*P* = 0.02). Therefore, the final MADRS scores reported were related to different (not specified) time points. Gałuszko-We¸gielnik et al^33^ did not present remission or response rates.

In the other retrospective study,^34^ patients with UDwP underwent a single administration of esketamine 0.5 mg/kg (either i.v. or s.c.) and were compared with matched controls with treatment-resistant unipolar depression without psychotic features (TRUD) treated with a single dose of i.v. esketamine 0.25 mg/kg. The outcome measure used for efficacy was ‘clinically meaningful change’ at 24 h post-dose, defined as a minimum decrease of 10 points on the MADRS, which occurred in 10 (66%) of those with UDwP and 22 (73%) of those with TRUD. These translated into response and remission rates at 24 h of 33% and 13% in the UDwP group and 40% and 23% in the TRUD group, respectively. The mean scores at 24 h and the mean change in scores were not disclosed. However, patients with UDwP had higher baseline MADRS scores [40.9 (s.d. 8.33) *v*. 33.6 (s.d. 5.36) in the TRUD group; *P* = 0.004], with no other controlled variables differing significantly between groups (age, sex, years with diagnosis). Furthermore, an exploratory analysis found no statistically significant difference between groups in the odds of a clinically meaningful change after treatment. Worsening of depressive symptoms was observed in two (13%) of the patients with UDwP and two (6%) of the patients with TRUD.

In the case reports and case series, depressive symptom reduction was widely reported, except in one of the case reports that included a patient with UDwP and catatonic features.^42^ In this case, it was mentioned that the patient remained depressed but showed improvement in the catatonic symptoms, 1 week after two 0.5 mg/kg ketamine infusions. In the remaining cases, depressive symptom score reduction varied from −94.4% 24 h after a single dose of 0.5 mg/kg esketamine to −47.1% 9 months of starting a 3-month intranasal esketamine protocol. Carter et al^37^ found a consistent reduction in Quick Inventory of Depressive Symptomatology (QIDS) scores from the second session (18 points) until the 12th session (5 points), with small increases to 8 and 7 points in the last two sessions, 3 months after starting treatment. Of the five cases of UDwP in which psychometric assessment of depressive symptoms was conducted, four patients showed clinical response from 24 h to 6 weeks, and three met criteria for remission within the same time frame. These response and remission rates were calculated on the basis of the provided psychometric data, according to consensual cut-off levels: for response, a score decrease of 50% or more; and for remission, scores of ≤9 on the MADRS, ≤5 on the QIDS or ≤7 on the Hamilton Rating Scale for Depression.^43–45^

#### Bipolar depression with psychotic symptoms

The study by Gałuszko-We¸gielnik et al^33^ included a group of patients (*n* = 18) with treatment-resistant BDwP who also underwent treatment with 0.5 mg/kg i.v. ketamine twice per week for 4 weeks. Similar to the UDwP group, the patients with BDwP showed a mean change of −14.9 points (s.d. 8.50) in MADRS scores after 5 weeks. Apart from that study, only one case series^40^ presented an individual with BDwP, who received a single 0.5mg/kg dose of esketamine s.c. His MADRS score decreased from 42 points to 7 points, 24 h after dosing. In addition, the patient reported persisting remission after 2 weeks.

### Safety

A summary of adverse events and psychotic symptoms reported in the included studies is presented in Table 3.

#### Psychotic symptoms

Overall, no study reported aggravation of psychotic symptoms. However, several did not assess or report these symptoms.^34,39,41^ The studies that measured psychotic symptoms with instruments^33,40^ did so with the BPRS,^46^ which is an 18-item instrument that measures not only psychotic symptoms but also symptoms related to mood, anxiety and motor behaviour. Each item is rated from 0 to 7, with higher scores reflecting increasing severity.

Gałuszko-We¸gielnik et al^33^ presented sum scores for the following four BPRS items: suspiciousness, hallucinations, unusual thought content and conceptual disorganisation. After 5 weeks, in both the UDwP and BDwP groups, scores fell from mean values of 7.2 (s.d. 2.2) and 7.3 (s.d. 2.6) to 0.0 (s.d. 0.0) and 0.4 (s.d. 1.5), respectively. All three patients described by Ajub and Lacerda^40^ showed reductions in BPRS total scores, which translated to a mean change 24 h post-treatment of −34.7 points (s.d. 9.1). In addition, remission lasted at least 2 weeks for patients 1 and 3 and at least 4 weeks for patient 2. Four of the six case reports^26,35–37^ mentioned remission of psychotic symptoms after treatment for up to 18 months. On the other hand, the case report by Trejos Orozco et al^42^ mentioned persistence of psychotic symptoms despite improvement in catatonic features 1 week after treatment.

#### Other adverse events

Ekstrand et al^41^ did not specifically report adverse events or discontinuation information for the subgroup of patients with UDwP. In the whole sample (*n* = 186), 93.4% of those who underwent ketamine treatment and 94.4% of those who underwent ECT reported at least one adverse event. In the ketamine group, 15.4% of adverse events were severe, with two cases classified as ‘very likely or probably caused by treatment’ but not detailed. In the ECT group, 25.6% of adverse events were severe, and 11 were considered as probably caused by treatment. Among the ketamine-treated patients, the median duration of most adverse events was 5 days or less. However, fatigue was reported for up to 27 days after treatment and dissociative symptoms and muscle pain for up to 11 days. The number of patients reporting long-lasting adverse events was significantly lower in the ketamine group. Discontinuation due to adverse events was reported in 13.2% and 4.4% of those treated with ketamine and ECT, respectively. Table 3 provides more details of the adverse symptoms and their duration.

Souza-Marques et al^34^ reported mild dissociative symptoms up to 2 h after dosing but did not specify incidence numbers. Gałuszko-We¸gielnik et al^33^ measured dissociative symptoms in both groups (UDwP and BDwP patients) with the Clinician Administered Dissociative States Scale. They observed an overall pattern of decreasing scores throughout treatment sessions, to mean scores of 0.2 (s.d. 0.6) in the UDwP group and 0.0 (s.d. 0.0) in the BDwP group, 1 week after the 4-week treatment. No other adverse events were reported in that study or by Carter et al.^37^ The remaining case reports and case series described the occurrence of nausea, light-headedness and dizziness, mild dissociation, derealisation, mild headache and fatigue; these symptoms were not reported as being the cause of treatment discontinuation.

In all studies, including the two that included patients with BDwP,^33,40^ there were no reports of affective switch to hypo(mania). According to Gałuszko-We¸gielnik et al,^33^ scores on the Young Mania Rating Scale did not significantly change during treatment with ketamine, nor did they differ significantly between BDwP patients and UDwP patients throughout the 5 weeks of follow-up.

### Risk of bias and quality assessment

The results of the bias and quality assessment are provided in Supplementary Tables 4 and 5. Of the included studies, only one was an RCT;^41^ the remaining studies were observational. The lack of randomisation in the observational studies introduced multiple potential biases, including selection and expectancy bias. This is particularly concerning in depression research, given well-documented high placebo response rates, which can confound results in non-randomised settings.^47^

Mischel et al^39^ and Trejos Orozco et al^42^ did not use instruments to assess depressive symptoms, and only Gałuszko-We¸gielnik et al^33^ and Ekstrand et al.^41^ assessed psychotic symptoms objectively (although the latter did not provide scores for the subgroup of patients with psychotic symptoms). Therefore, there was a significant lack of detailed data, especially regarding psychotic symptoms. In the study by Souza-Marques et al,^34^ patients were retrospectively divided into two groups (ECT and ketamine), and the ECT participants were chosen according to sex and age to closely match the ketamine group. Therefore, selection bias was probably introduced, and other confounding factors such as depression severity were not controlled for. In addition, although 5 weeks of follow-up, as in the study by Gałuszko-We¸gielnik et al,^33^ might have been enough to assess the effectiveness of ketamine treatment in the acute phase, it was insufficient to evaluate its effectiveness as a maintenance treatment or to detect late-onset adverse events. The study by Souza-Marques et al^33^ was further limited by its short 24-h follow-up. Ekstrand et al^41^ did not specify the incidence of adverse events or the number of patients who discontinued treatment prematurely for the subgroup of patients with psychotic symptoms, limiting the evaluation of adverse events in this subgroup.

Case reports and case series lacked representativeness, as expected; in addition, none specified how the diagnosis was confirmed. Furthermore, the follow-up duration was not sufficient to allow conclusions to be drawn regarding persisting effects or late-onset adverse events in the case reports byTrejos Orozco et al^42^ (follow-up of 1 week) and Ajub et al^40^ (follow-up of 2 weeks for two of the patients and 4 weeks for the other). Finally, Da Frota Ribeiro et al^35^ did not specify time points for symptom assessment, which limited their evaluation of treatment effectiveness over time.

## Discussion

To our knowledge, this is the first systematic review to focus on the efficacy and safety of ketamine and esketamine in patients with a diagnosis of depressive episode (either unipolar or bipolar) with psychotic symptoms. As a previously mentioned review featured patients with depressive symptoms in the context of various diagnoses with psychotic symptoms,^26^ the pool of patients with depression with psychotic symptoms treated with (es)ketamine has increased from five to 79 patients.

Despite the various regimens and formulations of ketamine enantiomers used in the different studies, there was an overall strong signal of antidepressant effects in both UDwP and BDwP patients. Across these diagnoses, MADRS mean score reductions ranged from −13.7 to −18.2 points in two of the three largest studies after up to 4 weeks of treatment.^33,41^ Observed remission ranged from 13% to 50% after 24 h to 4 weeks of treatment.^34,41^ Case series and case reports described depressive symptom score reductions that ranged from −70.9% 24 h after treatment to −47.1% 6 months after treatment. The mildest score reduction reported in these reports was −34.3%, 54 days after starting an intranasal esketamine regimen. The patient reported by Trejos Orozco et al^42^ showed an improvement in catatonic symptoms after 1 week, despite the persistence of depressive symptoms. However, the short duration of follow-up in this case, as well as in that of Souza-Marques et al^34^ (24 h), limited further conclusions regarding the course of these symptom changes. Notably, we did not find any studies reporting a lack of improvement in depressive symptoms, which suggests that publication bias may have inflated the perceived antidepressant effects. Nevertheless, one study reported deterioration of depressive symptoms 24 h after treatment with esketamine in two of the 15 patients with UDwP.^34^

None of the studies provided psychotherapeutic intervention either combined with (es)ketamine or as an alternative intervention. Only one study compared (es)ketamine with another intervention, in this case, ECT.^41^ The ECT group showed larger MADRS reductions than the ketamine group in patients with psychotic depression, although the difference was not statistically significant. However, it is not clear how other confounding factors might have influenced the results, as only age and symptom severity were compared between groups, and these factors did not significantly differ. In addition, there was a slightly higher number of sessions in the ECT group (including both patients with psychotic depression and those with non-psychotic depression), which might have also contributed to the greater treatment effectiveness. This highlights the need for more and larger studies to clarify how (es)ketamine treatment compares with other interventions in patients with these groups of disorders.

Another important finding of this review was the absence of reports of aggravating psychotic symptoms. Although there was an overall lack of objective measurement of psychotic symptoms, most of the included studies reported substantial qualitative improvements. This suggests that the risk of worsening psychosis may be lower than expected or even similar to the incidence in patients with depression without psychotic features. However, the absence of reports of worsening psychotic symptoms probably also reflects publication bias; we should be mindful of this when interpreting the limited evidence gathered so far.

During the screening phase, we found two conference abstracts of case reports which added to the evidence gathered, reporting significant antidepressant effects and absence of aggravation (or even improvement) of psychotic symptoms in patients with depressive episode.^48,49^ We also excluded studies that featured ketamine as an anaesthetic for ECT, given the potential confounding of the latter. However, a recent meta-analysis and a clinical trial on the use of ketamine in ECT in patients with mood disorders^50,51^ found no evidence of deterioration, and in some cases improvements were observed regarding psychotic symptoms (except in one study^52^ which reported hallucinations during recovery from anaesthesia that did not significantly differ from those in the placebo group).

We postulate that psychotic symptoms, being secondary to the primary mood disorder, improve alongside the antidepressant effects. However, the relationship between psychotic symptoms and the primary mood disorder is not yet clear.^2,53^ More recently, these symptoms have been shown to represent an independent symptom dimension of major depression that is not necessarily associated with depressive symptom severity.^54,55^

Despite uncertainties regarding the relationship between psychosis and depression, other studies following patients with primary psychotic disorders have shown that ketamine treatment may still be effective and safe. Similar to our findings, despite the possibly different underlying pathophysiology of psychotic symptoms in schizophrenia, no deterioration was found in patients with comorbid schizophrenia and depression after ketamine administration.^26^ In a trial that followed 50 patients with schizophrenia for an average of 8 months after undergoing ketamine treatment, there were transient increases in psychosis scores at 20 min post-infusion in both patients with schizophrenia and healthy participants, but these returned to baseline at 90 min and completely resolved after 3 h.^56^ Similar clues were found in a *post hoc* analysis of 12 patients with unipolar or bipolar depression who had a history of previous psychotic symptoms and were treated with a single dose of i.v. ketamine.^57^ The authors concluded that psychotic symptom scores did not differ from those of patients without a history of psychosis.

The prognostic value of the presence of psychotic symptoms in patients undergoing treatment with (es)ketamine is not clear. In the study by Pennybaker et al,^57^ the size of the antidepressant effects of ketamine was smaller for those with history of psychotic symptoms. Souza-Marques et al^34^ found no statistically significant difference between those with and without psychotic symptoms regarding the odds of a clinically meaningful change after treatment. However, they found that each one-point increase in baseline MADRS score predicted a consistent improvement of 0.82 points after treatment. A study (*n* = 409) that included 29 patients with a mood disorder and a history of psychosis found a trend (odds ratio = 2.75; *P* = 0.086) of patients with a history of psychosis showing higher response rates.^58^ Thus, having more severe depression and a history of psychosis might predict greater antidepressant effects from (es)ketamine treatment. These and other potential prognostic factors should be addressed in future studies. It should be noted that the relatively short duration of follow-up in most studies might not have been sufficient to detect potential deterioration of psychotic symptoms, keeping in mind that these findings must be compared with those from patients without psychotic symptoms or those receiving other treatments.

There was an overall lack of detailed reporting of adverse events in the groups of patients with psychotic symptoms in the larger studies.^33,34,41^ Similarly, there was a lack of safety data described in the case reports. Those that did report such data mentioned adverse effects that were mostly mild and congruent with those described in the existing literature.^59^ Although these data may suggest that patients with depressive episodes with psychotic symptoms are not more prone to different or more severe adverse events, the evidence is very limited and insufficient to draw conclusions about the safety of (es)ketamine in this population. Moreover, the identified publication bias implies that there are no safety data for cases in which the adverse events were intolerable or accompanied by a lack of symptom improvement.

We highlight the absence of reports of affective switches to hypo(mania). This absence is congruent with the low incidence (1.7%, *n* = 3) of (hypo)manic symptoms reported in a recent review of patients with bipolar depression treated with ketamine.^60^ Furthermore, the emergence of manic symptoms was reported either transiently during ketamine administration^60^ or between the third and fourth infusions.^10^ This suggests that despite the small sample (*n* = 18), the 5-week follow-up duration in the study by Gałuszko-We¸gielnik et al^33^ was enough to indicate that the incidence of manic symptoms is probably equally low in patients with BPwP.

We emphasise that a substantial proportion of the individuals included in these studies were under treatment with antidepressants, antipsychotics and/or mood stabilisers (see Supplementary Table 5). These drugs may have acted in parallel with or synergistically alongside (es)ketamine to reduce depressive and psychotic symptoms. In addition, the exacerbation of the latter may have been prevented by antipsychotic medication. Subgroup analysis on the basis of concomitant medication was not conducted in any study, and the influence of such medication on the outcomes must be taken into account, more so in the studies in which there were reports of recent treatment adjustment.^39,41,42^ On the other hand, this reflects similarity to a real-world scenario in which patients are often under multiple drug treatments. Nevertheless, future research should address how co-administration of different medications might influence outcomes. Regarding limitations in the review process, not including articles written in languages other than English might have induced selection bias.

Overall, the evidence body lacks adequate study designs for rigorous evaluation of the therapeutic benefits and harms of (es)ketamine in this population. In addition, the lack of representativeness, lack of homogeneous control groups and short follow-up duration contributed to a higher overall risk of bias. Furthermore, caution is advised when interpreting the limited existing evidence, as the lack of negative reports probably reflects publication bias, with negative results not being as appealing for publication.

Ketamine enantiomers seem to be well tolerated, fast-acting and effective in the improvement of depressive and psychotic symptoms, with no reports of worsening of the latter. Given that depression with psychotic features is already associated with worse prognosis and lacks evidence-based treatment alternatives, (es)ketamine may be considered for treatment-resistant cases as it is for depression without psychotic features. However, we highlight that the evidence is heterogeneous, lacks RCTs and is susceptible to multiple biases, particularly publication bias. Therefore, the safety and efficacy of (es)ketamine in individuals with depression with psychotic features remain unclear and require more rigorous research.

## Supporting information

Santos et al. supplementary material 1 Santos et al. supplementary material

Santos et al. supplementary material 2 Santos et al. supplementary material

## Data Availability

The studies included in this review are publicly available. The data extracted that support the findings of this study are available from H.C.S. and M.F.J. upon reasonable request.

## Supplementary material

The supplementary material is available online at https://doi.org/10.1192/bjo.2025.10830
