# Supplementary material for: (Es)ketamine as a treatment for depressive episodes with psychotic features: systematic review
Source: BJPsych Open. 2025 Oct 14;11(6):e240. doi: 10.1192/bjo.2025.10830 (PMC12529312; doi:10.1192/bjo.2025.10830)
Supplement: Santos et al. supplementary material 1 — Santos et al. supplementary material [file S2056472425108302sup001.docx]

| **Table S1 –** PRISMA for Abstracts Checklist | | | |
| --- | --- | --- | --- |
| **Section and Topic** | **Item #** | **Checklist item** | **Reported (Yes/No)** |
| **TITLE** | | |  |
| Title | 1 | Identify the report as a systematic review. | Yes |
| **BACKGROUND** | | |  |
| Objectives | 2 | Provide an explicit statement of the main objective(s) or question(s) the review addresses. | Yes |
| **METHODS** | | |  |
| Eligibility criteria | 3 | Specify the inclusion and exclusion criteria for the review. | No (Only broad inclusion criteria) |
| Information sources | 4 | Specify the information sources (e.g. databases, registers) used to identify studies and the date when each was last searched. | Yes |
| Risk of bias | 5 | Specify the methods used to assess risk of bias in the included studies. | No (specified in the main manuscript) |
| Synthesis of results | 6 | Specify the methods used to present and synthesise results. | No (specified in the main manuscript) |
| **RESULTS** | | |  |
| Included studies | 7 | Give the total number of included studies and participants and summarise relevant characteristics of studies. | Yes |
| Synthesis of results | 8 | Present results for main outcomes, preferably indicating the number of included studies and participants for each. If meta-analysis was done, report the summary estimate and confidence/credible interval. If comparing groups, indicate the direction of the effect (i.e. which group is favoured). | Yes |
| **DISCUSSION** | | |  |
| Limitations of evidence | 9 | Provide a brief summary of the limitations of the evidence included in the review (e.g. study risk of bias, inconsistency and imprecision). | No (specified in the main manuscript) |
| Interpretation | 10 | Provide a general interpretation of the results and important implications. | Yes |
| **OTHER** | | |  |
| Funding | 11 | Specify the primary source of funding for the review. | N/A |
| Registration | 12 | Provide the register name and registration number. | Yes |

| **Table S2 –** PRISMA Checklist | | | |
| --- | --- | --- | --- |
| **Section and Topic** | **Item #** | **Checklist item** | **Location where item is reported** |
| **TITLE** | | |  |
| Title | 1 | Identify the report as a systematic review. | Title |
| **ABSTRACT** | | |  |
| Abstract | 2 | See the PRISMA 2020 for Abstracts checklist. | See Table S1 |
| **INTRODUCTION** | | |  |
| Rationale | 3 | Describe the rationale for the review in the context of existing knowledge. | Pages 3 and 4 |
| Objectives | 4 | Provide an explicit statement of the objective(s) or question(s) the review addresses. | Page 4 |
| **METHODS** | | |  |
| Eligibility criteria | 5 | Specify the inclusion and exclusion criteria for the review and how studies were grouped for the syntheses. | Pages 5 and 6 |
| Information sources | 6 | Specify all databases, registers, websites, organisations, reference lists and other sources searched or consulted to identify studies. Specify the date when each source was last searched or consulted. | Page 4 |
| Search strategy | 7 | Present the full search strategies for all databases, registers and websites, including any filters and limits used. | Page 4 |
| Selection process | 8 | Specify the methods used to decide whether a study met the inclusion criteria of the review, including how many reviewers screened each record and each report retrieved, whether they worked independently, and if applicable, details of automation tools used in the process. | Pages 3 and 4 |
| Data collection process | 9 | Specify the methods used to collect data from reports, including how many reviewers collected data from each report, whether they worked independently, any processes for obtaining or confirming data from study investigators, and if applicable, details of automation tools used in the process. | Page 4 |
| Data items | 10a | List and define all outcomes for which data were sought. Specify whether all results that were compatible with each outcome domain in each study were sought (e.g. for all measures, time points, analyses), and if not, the methods used to decide which results to collect. | Page 5 |
|  | 10b | List and define all other variables for which data were sought (e.g. participant and intervention characteristics, funding sources). Describe any assumptions made about any missing or unclear information. | Page 5 |
| Study risk of bias assessment | 11 | Specify the methods used to assess risk of bias in the included studies, including details of the tool(s) used, how many reviewers assessed each study and whether they worked independently, and if applicable, details of automation tools used in the process. | Page 4 and 5 |
| Effect measures | 12 | Specify for each outcome the effect measure(s) (e.g. risk ratio, mean difference) used in the synthesis or presentation of results. | Page 6 |
| Synthesis methods | 13a | Describe the processes used to decide which studies were eligible for each synthesis (e.g. tabulating the study intervention characteristics and comparing against the planned groups for each synthesis (item #5)). | Page 6 |
|  | 13b | Describe any methods required to prepare the data for presentation or synthesis, such as handling of missing summary statistics, or data conversions. | Page 6 |
|  | 13c | Describe any methods used to tabulate or visually display results of individual studies and syntheses. | Page 6 |
|  | 13d | Describe any methods used to synthesize results and provide a rationale for the choice(s). If meta-analysis was performed, describe the model(s), method(s) to identify the presence and extent of statistical heterogeneity, and software package(s) used. | Page 6 |
|  | 13e | Describe any methods used to explore possible causes of heterogeneity among study results (e.g. subgroup analysis, meta-regression). | Page 6 |
|  | 13f | Describe any sensitivity analyses conducted to assess robustness of the synthesized results. | Not applicable |
| Reporting bias assessment | 14 | Describe any methods used to assess risk of bias due to missing results in a synthesis (arising from reporting biases). | Not applicable |
| Certainty assessment | 15 | Describe any methods used to assess certainty (or confidence) in the body of evidence for an outcome. | Not applicable |
| **RESULTS** | | |  |
| Study selection | 16a | Describe the results of the search and selection process, from the number of records identified in the search to the number of studies included in the review, ideally using a flow diagram. | Figure 1 |
|  | 16b | Cite studies that might appear to meet the inclusion criteria, but which were excluded, and explain why they were excluded. | Supplementary Material |
| Study characteristics | 17 | Cite each included study and present its characteristics. | Table 1 |
| Risk of bias in studies | 18 | Present assessments of risk of bias for each included study. | Pages 10 and 11 and Tables S3 and S4 |
| Results of individual studies | 19 | For all outcomes, present, for each study: (a) summary statistics for each group (where appropriate) and (b) an effect estimate and its precision (e.g. confidence/credible interval), ideally using structured tables or plots. | Tables 2 and 3 |
| Results of syntheses | 20a | For each synthesis, briefly summarise the characteristics and risk of bias among contributing studies. | N/A |
|  | 20b | Present results of all statistical syntheses conducted. If meta-analysis was done, present for each the summary estimate and its precision (e.g. confidence/credible interval) and measures of statistical heterogeneity. If comparing groups, describe the direction of the effect. | N/A |
|  | 20c | Present results of all investigations of possible causes of heterogeneity among study results. | Page 10 |
|  | 20d | Present results of all sensitivity analyses conducted to assess the robustness of the synthesized results. | N/A |
| Reporting biases | 21 | Present assessments of risk of bias due to missing results (arising from reporting biases) for each synthesis assessed. | N/A |
| Certainty of evidence | 22 | Present assessments of certainty (or confidence) in the body of evidence for each outcome assessed. | N/A |
| **DISCUSSION** | | |  |
| Discussion | 23a | Provide a general interpretation of the results in the context of other evidence. | Pages 11-13 |
|  | 23b | Discuss any limitations of the evidence included in the review. | Pages 11-13 |
|  | 23c | Discuss any limitations of the review processes used. | Page 13 |
|  | 23d | Discuss implications of the results for practice, policy, and future research. | Pages 11-13 |
| **OTHER INFORMATION** | | |  |
| Registration and protocol | 24a | Provide registration information for the review, including register name and registration number, or state that the review was not registered. | Page 14 |
|  | 24b | Indicate where the review protocol can be accessed, or state that a protocol was not prepared. | Page 14 |
|  | 24c | Describe and explain any amendments to information provided at registration or in the protocol. | Page 14 |
| Support | 25 | Describe sources of financial or non-financial support for the review, and the role of the funders or sponsors in the review. | Page 14 |
| Competing interests | 26 | Declare any competing interests of review authors. | Page 14 |
| Availability of data, code and other materials | 27 | Report which of the following are publicly available and where they can be found: template data collection forms; data extracted from included studies; data used for all analyses; analytic code; any other materials used in the review. | Page 15 |

# Search strings used for various platforms

Pubmed, OVID and Web of Science (Clarivate Analytics) databases were searched from inception until the 2^nd^ November 2023. The Web of Science’s Core Collection (1900-present), KCI-Korean Journal Database (1980-present), MEDLINE (1950-present), Preprint Citation Index (1991-present), ProQuest Dissertations & Thesis Citation Index (1637-present) and SciELO Citation Index databases (2002-present) were consulted, as well as the Cochrane Central Register of Reviews, and Ovid’s PsycINFO (1806 to October week 4 2023) and Embase (1974 to 2023 November 02) databases.

The following search terms were applied: (“ketamine” OR “esketamine” OR “*ketamine”) AND (“depress*” OR “bipolar”) AND (“psychosis” OR “psychotic”).

# List of all included papers

1. Ekstrand J, Fattah C, Persson M, Cheng T, Nordanskog P, Åkeson J, et al. Racemic Ketamine as an Alternative to Electroconvulsive Therapy for Unipolar Depression: A Randomized, Open-Label, Non-Inferiority Trial (KetECT). Int J Neuropsychopharmacol. 2022 May 1;25(5):339–49.
2. Souza-Marques B, Telles M, Leal GC, Faria-Guimarães D, Correia-Melo FS, Jesus-Nunes AP, et al. Esketamine for Unipolar Major Depression With Psychotic Features: A Retrospective Chart Review and Comparison With Nonpsychotic Depression. J Clin Psychopharmacol. 2022;42(4):408–12.
3. Gałuszko-Wȩgielnik M, Jakuszkowiak-Wojten K, Wiglusz MS, Cubała WJ, Pastuszak M. Central nervous system-related safety and tolerability of add-on ketamine to standard of care treatment in treatment-resistant psychotic depression in patients with major depressive disorder and bipolar disorder. Front Neurosci. 2023;17.
4. Ajub E, Lacerda ALT. Efficacy of Esketamine in the Treatment of Depression With Psychotic Features: A Case Series. Biol Psychiatry. 2018;83(1):e15–6.
5. Mischel N, Bjerre-Real C, Komisar J, Ginsberg B, Szabo ST, Preud’Homme X. Intravenous Ketamine Relieves Pain and Depression after Traumatic Suicide Attempts: A Case Series. J Clin Psychopharmacol. 2018 Apr;38(2):149–50.
6. da Frota Ribeiro CM, Sanacora G, Hoffman R, Ostroff R. The Use of Ketamine for the Treatment of Depression in the Context of Psychotic Symptoms: To the Editor. Biol Psychiatry. 2016;79(9):e65–6.
7. Trejos Orozco EA, Gutiérrez Segura JC, Ochoa Orozco SA. Safety and efficacy of Ketamine in a patient with psychotic depression, cardiovascular disease, and starvation risk. Rev Médica Risaralda. 2022 Sep 17;28(1).
8. Carter M, Solsrud K, Mischel N. Case report: Intranasal esketamine for severe major depressive disorder with psychotic features. Front Psychiatry. 2022;13(2).
9. Veraart JKE, Kamphuis J, Schlegel M, Schoevers RA. Oral S-ketamine effective after deep brain stimulation in severe treatment-resistant depression and extensive comorbidities. BMJ Case Rep. 2021;14(1):2020–2.
10. Zarrinnegar P, Kothari J, Cheng K. Successful Use of Ketamine for the Treatment of Psychotic Depression in a Teenager. J Child Adolesc Psychopharmacol. 2019;29(6):472–3.

# List of full-text screened reports that were excluded

## Reports excluded due to only including patients with depression without psychotic symptoms (n=19)

1. Lapidus KAB, Levitch CF, Perez AM, Brallier JW, Parides MK, Soleimani L, et al. A Randomized Controlled Trial of Intranasal Ketamine in Major Depressive Disorder. Biol Psychiatry. 2014 Dec;76(12):970–6.

2. Williamson D, Turkoz I, Wajs E, Singh JB, Borentain S, Drevets WC. Adverse Events and Measurement of Dissociation After the First Dose of Esketamine in Patients With TRD. Int J Neuropsychopharmacol. 2023 Mar 22;26(3):198–206.

3. Berman RM, Cappiello A, Anand A, Oren DA, Heninger GR, Charney DS, et al. Antidepressant effects of ketamine in depressed patients. Biol Psychiatry. 2000 Feb;47(4):351–4.

4. Lara DR, Bisol LW, Munari LR. Antidepressant, mood stabilizing and procognitive effects of very low dose sublingual ketamine in refractory unipolar and bipolar depression. Int J Neuropsychopharmacol. 2013 Oct 1;16(9):2111–7.

5. Pathak U, Ahuja SK, Dwivedi R, Mishra N, Kumar P, Mishra DK, et al. Antisuicidal efficacy of ketamine infusion in suicidal patients of depressive disorder. Indian J Psychiatry. 2021 Sep;63(5):483–9.

6. Milak MS, Rashid R, Dong Z, Kegeles LS, Grunebaum MF, Ogden RT, et al. Assessment of Relationship of Ketamine Dose With Magnetic Resonance Spectroscopy of Glx and GABA Responses in Adults With Major Depression: A Randomized Clinical Trial. JAMA Netw Open. 2020 Aug 12;3(8):e2013211.

7. Włodarczyk A, Cubała WJ, Gałuszko-Węgielnik M, Szarmach J. Central nervous system-related safety and tolerability of add-on ketamine to antidepressant medication in treatment-resistant depression: focus on the unique safety profile of bipolar depression. Ther Adv Psychopharmacol. 2021 Jan;11:20451253211011021.

8. Włodarczyk A, Cubała WJ, Gałuszko-Węgielnik M, Szarmach J. Dissociative symptoms with intravenous ketamine in treatment-resistant depression exploratory observational study. Medicine (Baltimore). 2021 Jul 23;100(29):e26769.

9. Vidal S, Gex-Fabry M, Bancila V, Michalopoulos G, Warrot D, Jermann F, et al. Efficacy and Safety of a Rapid Intravenous Injection of Ketamine 0.5 mg/kg in Treatment-Resistant Major Depression: An Open 4-Week Longitudinal Study. J Clin Psychopharmacol. 2018 Dec;38(6):590–7.

10. Pennybaker SJ, Luckenbaugh DA, Park LT, Marquardt CA, Zarate CA. Ketamine and Psychosis History: Antidepressant Efficacy and Psychotomimetic Effects Postinfusion. Biol Psychiatry. 2017;82(5):e35–6.

11. Farmer CA, Gilbert JR, Moaddel R, George J, Adeojo L, Lovett J, et al. Ketamine metabolites, clinical response, and gamma power in a randomized, placebo-controlled, crossover trial for treatment-resistant major depression. Neuropsychopharmacology. 2020 Jul 1;45(8):1398–404.

12. Wan LB, Levitch CF, Perez AM, Brallier JW, Iosifescu DV, Chang LC, et al. Ketamine Safety and Tolerability in Clinical Trials for Treatment-Resistant Depression. J Clin Psychiatry. 2015 Mar 25;76(03):247–52.

13. Fancy F, Rodrigues NB, Di Vincenzo JD, Chau EH, Sethi R, Husain MI, et al. Real‐world effectiveness of repeated ketamine infusions for treatment‐resistant bipolar depression. Bipolar Disord. 2023 Mar;25(2):99–109.

14. Zarate CA, Brutsche N, Laje G, Luckenbaugh DA, Venkata SLV, Ramamoorthy A, et al. Relationship of Ketamine’s Plasma Metabolites with Response, Diagnosis, and Side Effects in Major Depression. Biol Psychiatry. 2012 Aug;72(4):331–8.

15. Mathew SJ, Murrough JW, Aan Het Rot M, Collins KA, Reich DL, Charney DS. Riluzole for relapse prevention following intravenous ketamine in treatment-resistant depression: a pilot randomized, placebo-controlled continuation trial. Int J Neuropsychopharmacol. 2010 Feb;13(1):71–82.

16. Aan Het Rot M, Collins KA, Murrough JW, Perez AM, Reich DL, Charney DS, et al. Safety and Efficacy of Repeated-Dose Intravenous Ketamine for Treatment-Resistant Depression. Biol Psychiatry. 2010 Jan;67(2):139–45.

17. Rodrigues NB, McIntyre RS, Lipsitz O, Lee Y, Cha DS, Nasri F, et al. Safety and tolerability of IV ketamine in adults with major depressive or bipolar disorder: results from the Canadian rapid treatment center of excellence. Expert Opin Drug Saf. 2020 Aug 2;19(8):1031–40.

18. Grande LA. Sublingual Ketamine for Rapid Relief of Suicidal Ideation. Prim Care Companion CNS Disord

19. Vitek G, Langenfeld R, Walters RW, Elson A, Driscoll D, Ramaswamy S. Therapeutic and Safety Outcomes of Intravenous Ketamine for Treatment-refractory Depression in a Veteran Population: A Case Series. Mil Med. 2023 Jul 22;188(7–8):e2242–8.

## Report excluded due to referring to a case of post-schizophrenia depression

20. Bartova L, Papageorgiou K, Milenkovic I, Dold M, Weidenauer A, Willeit M, et al. Rapid antidepressant effect of S-ketamine in schizophrenia. Eur Neuropsychopharmacol. 2018 Aug;28(8):980–2.

## Reports excluded for having a mixed sample of patients with depression with and without psychotic symptoms, but without specific data for those with psychotic symptoms

21. Sharma RK, Kulkarni G, Kumar CN, Arumugham SS, Sudhir V, Mehta UM, et al. Antidepressant effects of ketamine and ECT: A pilot comparison. J Affect Disord. 2020 Nov;276:260–6.

## 22. Oliver PA, Snyder AD, Feinn R, Malov S, McDiarmid G, Arias AJ. Clinical Effectiveness of Intravenous Racemic Ketamine Infusions in a Large Community Sample of Patients With Treatment-Resistant Depression, Suicidal Ideation, and Generalized Anxiety Symptoms. J Clin Psychiatry. 2022 Sep 12;83(6):22–7.

## Report excluded due to review design

23. Galletly C. Look who’s talking? Aust N Z J Psychiatry. 2016 Nov;50(11):1031–2.

## Reports excluded for being conference proceedings

24. Bartova L, Papageorgiou K, Milenkovic I, Dold M, Weidenauer A, Willeit M, et al. A case report of rapid anti-suicidal and robust antidepressant effects of ketamine in post-psychotic depression. Eur Neuropsychopharmacol. 2019;29:S384–5.

25. A non-competitive nmda antagonist AZD6765 compared with ketamine and placebo on pharmaco-mri and cognitive mechanistic biomarkers in untreated major depressive disorder; a randomized double-blind controlled trial. Neuropsychopharmacology. 2012 Dec;38(S1):S198–313.

26. Olazabal N, Bustamante S, Madrazo M, Priego G, Osa L, Catalan A, et al. Intravenous ketamine for depression and congruent psychotic symptomatology: description of two consecutive cases. Eur Neuropsychopharmacol. 2017 Oct;27:S854.

27. Deakin J, Williams S, Downey D, McKie S, Goodwin G, Harmer C, et al. P.2.c.024 PharmacoMRI and cognitive effects of the potential antidepressant AZD6765 compared with ketamine in untreated major depressive disorder. Eur Neuropsychopharmacol. 2012 Oct;22:S264.

## Report excluded due to overlapping sample of with a more recent article included in the present review

28. Gałuszko-Węgielnik M, Chmielewska Z, Jakuszkowiak-Wojten K, Wiglusz MS, Cubała WJ. Ketamine as Add-On Treatment in Psychotic Treatment-Resistant Depression. Brain Sci. 2023;13(1).

Report excluded due to lack of longitudinal data

29. Laird SM, Sage M. Psychosis and ketamine. BMJ. 1972 Jan 22;1(5794):246–246.

# Quality Assessment

| **Table S3 –** Quality and bias assessment of the cohort studies and randomized controlled trial | | | | | | | | | |
| --- | --- | --- | --- | --- | --- | --- | --- | --- | --- |
| **Study** | **Newcastle-Ottawa for Cohort studies** | | | | | | | | |
|  | **Selection 1** | **Selection 2** | **Selection 3** | **Selection 4** | **Comparability 1** | **Outcome 1** | **Outcome 2** | **Outcome 3** | **Total** |
| Souza-Marques, B et al. 2022 | b)* | b) | a)* | a)* | c) | b)* | b) | a)* | Selection: 3 out of 4*  Comparability: 0 out of 2*  Outcome: 2 out of 3 * |
| Gałuszko-Wȩgielnik, M et al. 2023 | b)* | b) | a)* | a)* | c) | b)* | b) | a)* | Selection: 3 out of 4*  Comparability: 0 out of 2*  Outcome: 2 out of 3 * |
|  | **Cochrane risk-of-bias tool for randomized trials (RoB 2)** | | | | | |  |  |  |
|  | **Domain 1 RoB judgement** | **Domain 2 RoB judgement** | **Domain 3 RoB judgement** | **Domain 4 RoB judgement** | **Domain 5 RoB judgement** | **Overall RoB judgement** |  |  |  |
| Ekstrand, J et al. 2022 | Low risk | High risk | Low risk | High risk | Low risk | High risk |  |  |  |
| RoB: Risk Of Bias; Cochrane risk-of-bias tool for randomized trials (RoB 2) domains – Domain 1: Risk of bias arising from the randomization process; Domain 2: Risk of bias due to deviations from the intended interventions; Domain 3: Missing outcome data; Domain 4: Risk of bias in measurement of the outcome; Domain 5: Risk of bias in selection of the reported result | | | | | | |  |  |  |

| **Table S4** – Quality and bias assessment of the case reports and case series | | | | | | | | |
| --- | --- | --- | --- | --- | --- | --- | --- | --- |
| **Instrument** | **Items** | Ajub, E et al. 2017 | Mischell, N et al. 2018 | da Frota Ribeiro, C et al. 2016 | Trejos Orozco, E et al. 2022 | Carter, M et al. 2022 | Veraart, J. K. E et al. 2021 | Zarrinnegar, P et al. 2019 |
| **Modified Newcastle-Ottawa for case reports and case series** | **Q1** | NA | NA | NA | NA | NA | NA | NA |
|  | **Q2** | No | No | No | No | No | No | No |
|  | **Q3** | No | No | No | No | Yes | No | No |
|  | **Q4** | No | No | No | No | No | Yes | No |
|  | **Q5** | Yes | No | No | No | No | No | No |
|  | **Qualitative assessment** | Poor | Poor | Poor | Poor | Poor | Poor | Poor |
| **JBI for Case reports** | **1** |  | Yes | Yes | Yes | Yes | Yes | No |
|  | **2** |  | No | No | No | No | No | No |
|  | **3** |  | Yes | Yes | No | Yes | No | No |
|  | **4** |  | Unclear | No | No | No | No | No |
|  | **5** |  | Yes | No | Yes | Yes | No | Yes |
|  | **6** |  | No | Yes | No | Yes | No | Yes |
|  | **7** |  | No | No | No | Yes | Unclear | Yes |
|  | **8** |  | Yes | Yes | Yes | Yes | Yes | Yes |
| **JBI for Case Series** | **1** | No |  |  |  |  |  |  |
|  | **2** | No |  |  |  |  |  |  |
|  | **3** | Unclear |  |  |  |  |  |  |
|  | **4** | No |  |  |  |  |  |  |
|  | **5** | No |  |  |  |  |  |  |
|  | **6** | No |  |  |  |  |  |  |
|  | **7** | No |  |  |  |  |  |  |
|  | **8** | Yes |  |  |  |  |  |  |
|  | **9** | NA |  |  |  |  |  |  |
|  | **10** | NA |  |  |  |  |  |  |
|  | **Questions addressed with the Modified Newcastle-Ottawa for case reports and case series:** 1. Did the patient(s) represent the whole case(s) of the medical center? 2. Was the diagnosis correctly made? 3. Was the other important diagnosis excluded? 4. Were all the important data cited in the report? 5. Was the outcome correctly ascertained?  **Questions addressed with the JBI for Case Reports:** 1. Were patient’s demographic characteristics clearly described? 2. Was the patient’s history clearly described and presented as a timeline? 3. Was the current clinical condition of the patient on presentation clearly described? 4. Were diagnostic tests or assessment methods and the results clearly described? 5. Was the intervention(s) or treatment procedure(s) clearly described? 6. Was the post-intervention clinical condition clearly described? 7. Were adverse events (harms) or unanticipated events identified and described? 8. Does the case report provide takeaway lessons?  **Questions addressed with the JBI for Case Series:** 1. Were there clear criteria for inclusion in the case series? 2. Was the condition measured in a standard, reliable way for all participants included in the case series? 3. Were valid methods used for identification of the condition for all participants included in the case series? 4. Did the case series have consecutive inclusion of participants? 5. Did the case series have complete inclusion of participants? 6. Was there clear reporting of the demographics of the participants in the study? 7. Was there clear reporting of clinical information of the participants? 8. Were the outcomes or follow up results of cases clearly reported? 9. Was there clear reporting of the presenting site(s)/clinic(s) demographic information? 10. Was statistical analysis appropriate? | | | | | | | |

| **Table S5** – Other clinical data of the patients in the included articles | | | | | | | | | | |
| --- | --- | --- | --- | --- | --- | --- | --- | --- | --- | --- |
| **First author** | **N** | **%/n of Substance use Disorders** | **%/n of Anxiety disorders** | **%/n of Primary Psychotic disorders** | **Other psych comorbidities** | **Non-psych comorbidities** | **n/(%) under antidepressants** | **n/(%) under antipsychotics** | **n/(%) under mood stabilizers** | **n/(%) under benzodiazepines** |
| Ekstrand, J et al. 2022 | 35 | 0 (exclusion criterion) | N/D | 0 (exclusion criterion) | N/D | N/D | ECT: 44%; Ketamine: 43%; N/D for the subgroup | ECT: 19%; Ketamine: 14%; N/D for the subgroup | ECT: 7%; Ketamine: 4%; N/D for the subgroup | N/D |
| Souza-Marques, B et al. 2022 | 15/45 | 0 | MDD w/ P: 0 (0%);  TRD-MDD: 8 (27%) with GAD and 10 (33%) with Panic disorder | 0 | MDD w/ P: 1 (7%) with Schizoid Personality Disorder and 1 (7%) with Obsessive-compulsive Personality disorder;  TRD-MDD 2 (13%) with OCD | N/D | N/D | N/D | N/D | N/D |
| Gałuszko-Wȩgielnik, M et al. 2023 | TR UDwP (n=17) | N/D | N/D | N/D | N/D | Hypertension (41.2%); Diabetes (5.9%); Epilepsy (5.9%); Hypercholesterolemia (17.6%); Other (23.5%) | Vast majority* | Aripiprazole (5.9%); Quetiapine (23.5%); Olanzapine (11.8%); Zuclopenthixol (5.9%) | Lithium (5.9%); Valproate (11.8%); Lamotrigine (17.6%) | Non disclosed |
|  | TR BDwP (n=18) |  |  |  |  | Hypertension (27.8%); Diabetes (5.6%); Epilepsy (16.7%); Hypercholesterolemia (5.6%); Other (33.3%) | Vast majority* | Aripiprazole (27.8%); Quetiapine (44.4%); Olanzapine (33.3%); Risperidone (5.6%) | Lithium (44.4%); Valproate (33.3%); Lamotrigine (38.9%) | Non disclosed |
| Ajub, E et al. 2017 | 3 of 4 | Patient 1 and 3 with Alcohol Use Disorder  (66.6%) | Patient 2 with Social Anxiety Disorder  (33.3%) | N/A | N/A | N/D | Patient 1: Sertraline 150mg; Patient 2: Sertraline 100mg;  (66.6%) | Patient 1: Risperidone 3mg; Patient 2: Quetiapine 100mg; Patient 3: Risperidone 3mg;  (100%) | 1 (33.3%) Patient 3: Lithium 600mg; Sodium Valproate 1000mg; Lamotrigine 50mg | 0 |
| Mischell, N et al. 2018 | 2 | N/D | Patient 1 had history of "anxiety" | N/D | N/D | Patient 1 had Esophageal cancer and Bigeminy | Patient 1: Venlafaxine + Mirtazapine;  Patient 2: Venlafaxine 225mg + Mirtazapine 30mg (both started during admission)  (100%) | (100%) Patient 1: Aripiprazole (interrupted during admission); Patient 2: Aripiprazole "at an antipsychotic dose" started during admission | 0% | 0% |
| da Frota Ribeiro, C et al. 2016 | 2 | N/D | N/D | Patient 2: Schizoaffective disorder | N/D | N/D | Patient 1: Venlafaxine 300mg/day | Patient 1: Ziprasidone 80mg/day;  Patient 2: Clozapine 225mg/day | Patient 2: Lamotrigine 200mg/day and Lithium 300mg/day; | Patient 1: Clonazepam 1mg/day |
| Trejos Orozco, E et al. 2022 | 1 | N/D | N/D | N/D | N/D | Patent foramen ovale; Atrial fibrillation | Sertraline (dose N/D) | Olanzapine (dose N/D) | 0 | Lorazepam (dose N/D) |
| Carter, M et al. 2022 | 1 | 0 | GAD | 0 | 0 | Asthma; Hypertension | Bupropion 150mg; Sertraline 50mg; Trazodone 100mg | Aripiprazole 10mg | 0 | Clonazepam 0.5mg |
| Veraart, J. K. E et al. 2021 | 1 | 0 | 0 | 0 | OCD with DBS implants | N/D | Venlafaxine 300mg | Clozapine 450mg | 0 | nitrazepam 5mg 3x/week |
| Zarrinnegar, P et al. 2019 | 1 | 0 | GAD | 0 | Post-traumatic stress disorder | N/D | Venlafaxine (dose N/D) | Ziprasidone (dose N/D) | 0 | 0 |
| **BDwP** – Bipolar Depression with Psychotic symptoms; **DBS**: Deep Brain Stimulation; **ECT** – Electroconvulsive Therapy; **GAD** – Generalized Anxiety Disorder; N**/D** – Not disclosed; **OCD** – Obsessive Compulsive Disorder; **SD** – Standard Deviation; **TR** – Treatment Resistant; **UD** – Unipolar Depression; **UDwP** – Unipolar Depression with Psychotic symptoms;  *the overall number of patients under antidepressants was not provided but the available data suggests most (if not all) were treated with antidepressant medication; | | | | | | | | | | |
